# Se@SiO_2_ nanocomposites suppress microglia-mediated reactive oxygen species during spinal cord injury in rats

**DOI:** 10.1039/c8ra01906a

**Published:** 2018-04-30

**Authors:** Weiheng Wang, Xiaodong Huang, Yongxing Zhang, Guoying Deng, Xijian Liu, Chunquan Fan, Yanhai Xi, Jiangming Yu, Xiaojian Ye

**Affiliations:** Department of Orthopaedics, Changzheng Hospital, Second Military Medical University No 415 Fengyang Road Shanghai 200003 China yjm_spine@smmu.edu.cn xiyanhai@smmu.edu.cn xjyespine@smmu.edu.cn +86 021 81870950 +86 021 81885624 +86 021 81886807 +86 021 81870952; Trauma Center of Shanghai General Hospital, Shanghai Jiaotong University School of Medicine Shanghai 201620 China; College of Chemistry and Chemical Engineering, Shanghai University of Engineering Science Shanghai 201620 China; Department of Orthopaedic Surgery, The 175th Hospital of PLA, Orthopaedics Center of PLA, Affiliated Southeast Hospital of Xiamen University Zhangzhou Fujian Province PR China

## Abstract

Selenium (Se) is an essential trace element with strong antioxidant activity, showing a great prospect in the treatment of spinal cord injury (SCI). However, the narrow gap between the beneficial and toxic effects has limited its further clinical application. In this experiment, we used porous Se@SiO_2_ nanocomposites (Se@SiO_2_) modified by nanotechnology as a new means of release control to investigate the anti-oxidative effect in SCI. *In vitro* Se@SiO_2_ toxicity, anti-oxidative and anti-inflammatory effects on microglia were assayed. *In vivo* we investigated the protective effect of Se@SiO_2_ to SCI rats. Neurological function was evaluated by Basso, Beattie and Bresnahan (BBB). The histopathological analysis, microglia activation, oxidative stress, inflammatory factors (TNF-α, IL-1β and IL-6) and apoptosis were detected at 3 and 14 days after SCI. The favorable biocompatibility of Se@SiO_2_ suppressed microglia activation, which is known to be associated with oxidative stress and inflammation *in vivo* and *in vitro*. In addition, Se@SiO_2_ improved the rat neurological function and reduced apoptosis *via* caspase-3, Bax and Bcl-2 pathways in SCI. Se@SiO_2_ was able to treat SCI and reduce oxidative stress, inflammation and apoptosis induced by microglia activation, which may provide a novel and safe strategy for clinical application.

## Abstract 2

Selenium (Se) is an essential trace element with strong antioxidant activity, showing a great prospect in the treatment of spinal cord injury (SCI).

## Graphical abstract

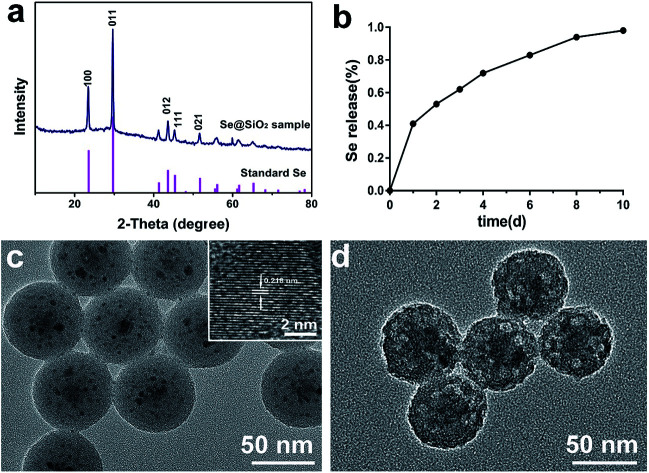

## Introduction

Spinal cord injury (SCI) is a serious clinical trauma with a high disability rate.^1^ With changes in social economy and lifestyle, the incidence of SCI is increasing annually.^2^ Patients with SCI often present with severe limb disorders and poor prognosis, resulting in severe economic and social burdens. The pathological mechanism of SCI is complex and remains poorly understood.^3^ A number of factors are involved in the development of SCI, including oxidative stress,^4,5^ inflammation^6^ and apoptosis.^7^ A large number of studies have shown that oxidative stress is an important factor in SCI. Primary traumatic SCI induces the release of reactive oxygen species (ROS) and inflammatory cytokines, resulting in the activation of microglia.^6^ Microglia are a type of glial cells located throughout the central nervous system (CNS) and have diverse important functions in response to environmental changes in the spinal cord.^8^ Excessive activation of microglia may induce the release of large amounts of ROS and inflammatory cytokines, leading to more serious secondary SCI.^9,10^ Oxidative stress is closely related to ROS.^11^ ROS can produce by cell lysis and oxidative burst,^12^ the presence of an excess of free transition metals^13^ and normal cellular respiration.^14^ There are free radical defense mechanisms in the CNS, mainly including superoxide dismutase (SOD) and other antioxidants, and chelation of transition metal catalysts.^15^ There is a balance between the production and clearance of free radicals. Microglia activation at the injured site of the spinal cord can produce large amounts of ROS, leading to imbalance of oxidative stress, which may further lead to lipid peroxidation of the cell membrane, degeneration of intracellular proteins and enzymes, DNA damage and neuronal death or apoptosis.^16^

Selenium (Se) is one of the essential micronutrients for human beings.^17^ It is also the critical catalyst for glutathione peroxidase (GPx)^18,19^ and mammalian thioredoxin reductase,^20^ two important anti-oxidant enzymes that can suppress oxidative stress. Se deficiency is associated with cancer, viral infections, and a variety of cardiovascular diseases, such as Keshan disease.^21^ Many studies have shown that daily intake of 200 mg Se can reduce the incidence of cancer remarkably.^22^ Se can reduce myocardial injury induced by oxidative stress, reduce the infarct size and improve ventricular remodeling after infarction by increasing the expression and activity of GPx in serum.^23,24^ Studies have shown that Se has a good neuroprotective effect by inhibiting ROS-mediated apoptosis in traumatic brain injury.^25,26^ Therefore, the use of antioxidant Se for the treatment of SCI has a promising prospect. Compared with other antioxidants, Se is more stable and economical. However, the direct use of pure Se (nano-scale or marco-scale) has many limitations in clinical treatment due to the narrow margin between the beneficial and toxic effects, which limited Se further application.^27,28^ To solve this problem, an effective way is to directly deliver Se to target organization through nanocarriers, which is expected to achieve sustained drugs release, depress the toxic side effects and simultaneously enhance the therapeutic efficiency.^29^ Various nanocarriers have been widely studied, such as liposomes,^30^ nanocapsules,^31^ dendrimers,^32^ gold nanostructures,^33^ silica nanostructures,^34,35^*etc.* However, silica nanostructures have unprecedented advantages such as good compatibility, adjustable pore structure, and simple, low-cost and controllable fabrication. Recently, Liu *et al.*^36^ reported the use of porous Se@SiO_2_ nanocomposites as a new strategy of nano-Se controlled release, which shown good biocompatibility for normal cells and could be used as drug carriers. Nano-modified Se could not only reduce the risk of Se toxicity^37,38^ but showed high biological activity both *in vivo* and *in vitro*.^39,40^ Some recent studies have proven that porous Se@SiO_2_ nanocomposites shown good safety and antioxidant effects. Zhu *et al.* found that porous Se@SiO_2_ nanospheres can be used to treat paraquat-induced acute lung injury by resisting oxidative stress.^41^ Deng *et al.* found that porous Se@SiO_2_ nanospheres can protect steroid-induced osteonecrosis of the femoral head^42^ and protect the femoral head from methylprednisolone-induced osteonecrosis by resisting oxidative stress.^43^ The use of porous silica nanocarriers to control the release of nano-Se could achieve a long-term effective concentration of Se, thus improve the therapeutic outcome.^42^

Porous Se@SiO_2_ nanocomposites are a new type of material with a good anti-oxidative effect and nano-Se controlled release, which represents a promising, safe and effective way for SCI treatments. However, its effect on SCI and related mechanisms have not been reported. The aim of this study was to research whether porous Se@SiO_2_ nanospheres could significantly alleviate oxidative stress and the inflammatory response of microglia-mediated during SCI in rats, and explored the underlying mechanism *in vitro* and *in vivo*.

## Materials and methods

### Synthesis and characterization of porous Se@SiO_2_ nanocomposites

Porous Se@SiO_2_ nanocomposites were prepared according to the previous method^36^ and provided by Shanghai University of Engineering Science (Shanghai, China). The surface characteristics of the prepared material were examined using D/max-2550 PC X-ray diffractometry (XRD; Rigaku, Cu-Kα radiation) and transmission electron microscopy (TEM; JEM-2100F). The cumulative release kinetics of Se elements in the porous Se@SiO_2_ and nanospheres were detected in PBS (0.01 M, pH 7.4). They were then dissolved in deionized water for subsequent experiments.

### Animals

Sprague-Dawley (SD) rats (female, 200–220 g) and suckling SD rats born within 24 h were purchased from the experimental animal center of the Second Military Medical University (Shanghai, China). Before the experiment, rats were housed in an experimental animal center at 20–22 °C without specific pathogens for 1–2 weeks to adapt to the environment, with free access to food and water. All animal experiments were approved by the animal ethics committee of the Second Military Medical University. All experiments were performed in compliance with the Regulations for the Administration of Affairs Concerning Experimental Animals in Shanghai.

### Culture and identification of primary microglia

Based on classical glial cell culture reported by McCarthy *et al.*,^44^ primary microglia were extracted using a modified shaking method. Briefly, the 24 h newborn SD rat was submerged in alcohol for 10 min for disinfection. The skull was stripped and placed in an ice-PBS buffer. The pia mater, blood vessels, hippocampus and midbrain were removed under a dissecting microscope. The isolated cortex was placed in trypsin, cut into small pieces and digested for 5 min. After digestion, the cells were repeatedly washed, dispersed into a single cell suspension and filtered through a 400 mesh membrane to prepare a whole brain single cell suspension. The single cell suspensions was re-suspended in DMEM/F12 medium and seeded in polylysine-coated T25 cells at 4 × 10^8^ per L cells. The cells were incubated in a 37 °C, 5% CO_2_ and saturated humidity incubator and shaken at 260 rpm at 37 °C for 2 h. The shaken-down cell suspension was collected and inoculated into a T25 flask. After 1 h conventional culture, the untreated cells were discarded to obtain purified microglia. Purity of the microglia was tested by Iba-1 immunofluorescence staining.

### Toxicity of porous Se@SiO_2_ nanocomposites on microglia *in vitro*

To investigate the effect of different concentrations of porous Se@SiO_2_ nanocomposites on microglia toxicity, primary microglia 5 × 10^3^ cells per well and 100 μl complete medium were inoculated into 96-well plates. After adherence, they were replaced with different intervention media: 0, 10, 20, 40, 80, 160 and 320 μg ml^−1^ porous Se@SiO_2_ nanocomposites and cultured for 24 h. Cell counting kit 8 (CCK-8, Biyuntian, China) was used in strict accordance with the test procedure instructions. Cytotoxicity of different concentrations of porous Se@SiO_2_ nanocomposites on microglia was examined using a microplate reader (Bio Tek, USA) at 450 nm. The experiment was repeated 3 times with 2 replicates per condition.

### Effects of Se@SiO_2_ on oxidative stress injury of microglia mediated by H_2_O_2_*in vitro*

First, the median lethal dose of H_2_O_2_ against microglia (median, lethal, dose, LD_50_) was detected, and this concentration was used in subsequent trials. Microglia 5 × 10^3^ cells per well in 100 μl complete medium were inoculated into 96-well plates and then incubated in DF-12 medium with out serum containing 0, 100, 200, 400, 600, 800 and 1000 μM H_2_O_2_ for 24 h. Cytotoxicity was measured using CCK-8. 400 μM H_2_O_2_ was selected as the treatment condition of microglia to induce oxidative stress. Based on the effect of different concentrations on the viability of microglia, 10 and 40 μg ml^−1^ Se@SiO_2_ were used to study the Se@SiO_2_ dose effect in this experiment. The primary microglia were cultured in 6-well plates at a density of 1 × 10^5^ per well for 12 h. After complete adherence of the microglia, the 6-well plates were divided into a blank, control, 10 μg ml^−1^ Se, and 40 μg ml^−1^ Se groups. The blank group was incubated for 24 h in a complete culture medium containing 0 μg ml^−1^ of porous Se@SiO_2_ nanocomposites. The control group was incubated for 24 h in a complete medium containing 400 μM H_2_O_2_. While the 10 μg ml^−1^ Se and 40 μg ml^−1^ Se groups were incubated for 24 h using 400 μM H_2_O_2_ and complete culture medium containing 10, 40 μg ml^−1^ porous Se@SiO_2_ nanocomposites, respectively. After 24 h, the supernatant and adherent cells were collected. CCK-8 assay was used to detect microglia viability. Malondialdehyde (MDA) and SOD kits were used to detect intracellular oxidative stress levels. TNF-α, IL-1β and IL-6 ELISA kits were used to detect cell supernatant inflammatory factors. The experiment was repeated 3 times with 2 replicates per condition.

### Establishment of the SCI rat model and implantation of the intrathecal catheter

The rat was anesthetized by pentobarbital sodium (50 mg kg^−1^, intraperitoneal injection) and fixed in a bayonet-type rat fixator. The intrathecal catheter implantation rat model was established as Yaksh and Rudy described.^45^ After successful anesthesia, the lamina was cut off from the lumbar region, and the PE-10 catheter was inserted. Effusion of cerebrospinal fluid (CSF) along the catheter was regarded as successful catheterization. Each rat was housed in a single cage. After full awakening, rats with motor dysfunction were removed and 10 μl 2% lidocaine was injected along the catheter. The presence of movement dysfunction in both lower extremities of the rat 30 seconds after injection indicates successful catheterization. After 1 day successful establishment of the intrathecal catheter implantation rat model, improved Allen SCI model of contusion was used with modifications as previously described.^46,47^ A 10 g weighted hammer fell from a height of 50 mm and impinged on rat thoracic 9–11 vertebrae. For the first 3 d after surgery, 50 000 units conventional penicillin was delivered *via* intramuscular injection to prevent infection. The rats received abdominal massages every 6 h after the procedure to help them urinate until they were capable of urinating by themselves.

### *In vivo* experimental design and sample collection

135 rats were randomly divided into a sham, control and Se@SiO_2_ group, with an average of 45 rats per group. After successful establishment of the model, porous Se@SiO_2_ nanocomposites (1 mg kg^−1^, 10 μl) or normal saline (NS, 10 μl) was injected intrathecally into Se@SiO_2_ and control groups. The concentration of porous Se@SiO_2_ nanocomposites was selected according to our previous research.^41,42^ Se@SiO_2_ (1 mg kg^−1^, 10 μl) or 10 μl NS was administered gently into the restrained conscious rats by using a microinjector (Gaoge, Shanghai, China) attached to the intrathecal catheter within 2 min, followed by a flush with 20 μl NS. Se@SiO_2_ and the vehicle were administered immediately following SCI and daily after surgery. 15 rats in each group were sacrificed with the overdose anesthesia after 3 and 14 days SCI respectively. The 3 mm around the center of the damaged spinal cord was sampled and collected. 5 rats in each group were sacrificed for western blotting analysis. 5 rats in each group were examined for MDA, SOD and inflammatory factors (TNF-α, IL-1β and IL-6). The remaining 5 rats in each group were subjected to histological and immunofluorescence assays.

### Histopathological observation of the damaged spinal cord segment

The spinal cord was fixed in 4% PFA, stained with hematoxylin-eosin (HE), sliced into 5 μm sections, and observed for morphological changes under a microscope after 3 and 14 days SCI respectively. The quantification percentage of cavity area were calculated 14 days after SCI (percentage of cavity area = cavity area/total area × 100%, *n* = 5).

### Detection of oxidative stress by SOD and MDA kits

To confirm the change in oxidative stress as represented by the content of SOD and MDA after Se@SiO_2_ treatment, the injured spinal cord tissue and Se@SiO_2_-treated microglia were collected and tested by the SOD and MDA kits, knowing that MDA is an indicator of lipid oxidation and SOD is an indicator of antioxidant ability. Then, adherent cells or 100 mg spinal tissue was homogenized immediately in 1 ml NS, and the homogenate was centrifuged at 2000 g for 15 min at 4 °C. The protein concentration in the supernatant was determined by BCA assay, and the SOD activity (U m^−1^) and MDA concentration (nmol mg^−1^ protein) were measured using SOD and MDA kits (Nanjing Jiancheng Bioengineering Institute, Nanjing, China).

### Detection of inflammation by ELISA

To confirm changes in inflammatory factors (TNF-α, IL-1β and IL-6) after Se@SiO_2_ treatment, the injured spinal cord tissue and Se@SiO_2_-treated microglia were collected and tested by ELISA kits (R&D Systems, Minneapolis, MN, USA). 100 μg spinal tissue or microglia cell supernatant was homogenized immediately in 1 ml NS, and the homogenate was centrifuged at 2000 g for 15 min at 4 °C. The protein concentration in the supernatant was determined by BCA assay. ELISA kit manual operation tested in strict accordance to standard protocols and used the microplate reader (ELx800, BioTek) at 450 nm to calculate the content of the sample.

### Western blot

3 and 14 days after SCI, 3 mm spinal cord in the center of the lesion was collected, frozen and stored (*n* = 5). The samples were dissolved in the cell lysate and then centrifuged at 14 000 g at 4 °C for 15 min, and the protein concentration in the sample was determined by BCA. The protein concentration in each sample was adjusted to 50 μg. The sample was processed according to the regulations strictly. The primary antibody Iba-1 (1 : 500, Abcam ab5076, Cambridge, UK), caspase-3 (1 : 500, cell signaling #9662, USA), Bax (1 : 1000, Abcam ab32503, Cambridge, UK) and Bcl-2 (1 : 500, Abcam ab59348, Cambridge, UK) antibody were incubated overnight and then labeled with the corresponding secondary antibody (Jackson 1: 2000). The ratio of optical density of band to optical density of GAPDH band was calculated as the relative expression level of the sample protein level.

### Immunohistochemistry

3 and 14 days after SCI, the injured spinal card was harvested for immunohistochemistry to detect the expressions of Ibl-1 and neurofilament 200 (NF200). The rats were killed by overdose anesthesia and infused with pre-cooling PBS for 10 min firstly and then perfused with 4% PFA for 20 min. The harvested spinal cord was fixed in 4% PFA for 4 h, dehydrated with 20% sucrose for 24 h, and then dehydrated with 30% sucrose for 48 h. After tissue deposition, the tissue was fixed with OTC, sliced into 10 μm sections with a microtome, immunohistochemically stained, washed with PBS for 3 times, incubated overnight with primary antibodies anti-Iba-1 (1 : 200, Abcam, Cambridge, UK) and mouse anti-NF200 (1 : 200, Abcam, Cambridge, UK) at room temperature, washed again with PBS for 3 times, incubated with the corresponding secondary antibody for 2 h, dyed with dapi, and sealed with 50% PBS glycerol. Finally, images were captured with a fluorescence microscope (Olympus, Tokyo, Japan) by randomly selecting 10 fields at the spinal tissue. For quantification, the percentage of positive cells was calculated as (number of positive cells)/(total number of cells in the field) × 100%.

### Terminal deoxynucleotidyl transferase dUTP nick-end labeling (TUNEL) staining

After TUNEL staining 3 and 14 days after SCI, the number of apoptotic cells in the injured spinal card was quantified with a fluorescence detection kit (Roche Molecular Biochemicals, Indianapolis, IN, USA). The sections were de-paraffinized, re-hydrated at 60 °C, incubated in a 20 μg ml^−1^ proteinase K working solution for 15 min at room temperature following standard protocols, and observed under a microscope (Carl Zeiss Jena, Oberkochen, Germany). The number of apoptotic cells and the total number of cells were determined using Image-Pro Plus 6.0 (Media Cybernetics, Silver. Spring, USA). Ten random fields per section were analyzed and the apoptosis index was calculated (*n* = 5).

### Hind-limb motor function after SCI modeling in rats

45 rats were placed on the platform before and 1, 3, 7, 14, 21 and 28 d after modeling, and scored by the Basso, Beattie and Bresnahan (BBB) scale.^48^ The activities of the hip, knee, ankle and trunk movement and coordination were recorded by two technicians who were blind to the experimental grouping. The scores of two individuals were compared; if they were found to be of different, a joint evaluation was required (*n* = 15).

### Statistical analysis

SPSS 21 software (SPSS, Chicago, IL, USA) was used for data statistical analysis. Data were expressed as mean ± standard deviation (SD). The hind-limb movement and neurological function scores were analyzed by ANOVA and Fisher's LSD post hoc test. The other indexes were evaluated by ANOVA. *P* < 0.05 were considered statistically significant.

## Results

### Properties of porous Se@SiO_2_ nanocomposites

**Fig. 1:**
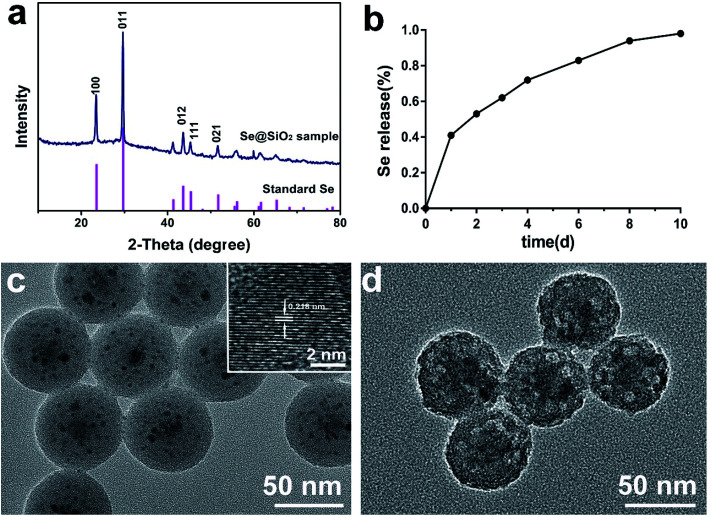
Characterization and Se cumulative release kinetics of the Se@SiO_2_ nanocomposites: (a) the XRD pattern of the solid Se@SiO_2_ nanocomposites and the standard hexagonal phase of Se (JCPDS card no: 65-1876). (b) The cumulative release kinetics of Se from the porous Se@SiO_2_ nanospheres in PBS (0.01 M, pH 7.4, 37 °C). (c) TEM image of the prepared solid Se@SiO_2_ nanocomposites (inset: HRTEM of a solid Se@SiO_2_ nanocomposite). (d) TEM image of the prepared Se@SiO_2_ nanocomposites.

A series of tests on XRD (Fig. 1a), cumulative release kinetics (Fig. 1b) and TEM (Fig. 1c and d) confirmed that the nanocomposites used in this test were porous Se@SiO_2_ nanocomposites with good Se controlled release. The structure of the nanocomposites was detected by XRD spectra, showing (100), (011), (110) and (012) clear characteristic peaks, which were the same as those of the hexagonal system referenced by the standard Se (JCPDS, card, no. 65–1876). The amorphous silica Se@SiO_2_ nanocomposites exhibited a stable increase in the low-angle region in the XRD spectra. TEM showed that the diameter of the homogeneous nanocomposites was about 50 nm. The dispersed nanoparticles had an interplanar spacing about 0.218 nm, which matched to the spacing of the standard hexagonal Se (110) crystal planes, further confirming that the small nanoparticles dispersed in the silica were Se nanometer crystals. The previous Brunauer–Emmett–Teller (BET) characterization showed that Se@SiO_2_ nanocomposites were porous.^36^ It was found that the pyrrolidinylquinoxaline (PVP) penetrated into the silica shell and formed a channel in the Se@SiO_2_ nanocomposite, through which very small nanoparticles were able to release from the porous Se@SiO_2_ nanocomposite material. The Se element in Se@SiO_2_ nanocomposites could achieve controlled release at 37 °C 0.01 M PBS (Fig. 1b).

### Morphology and purity of the primary microglia

**Fig. 2:**
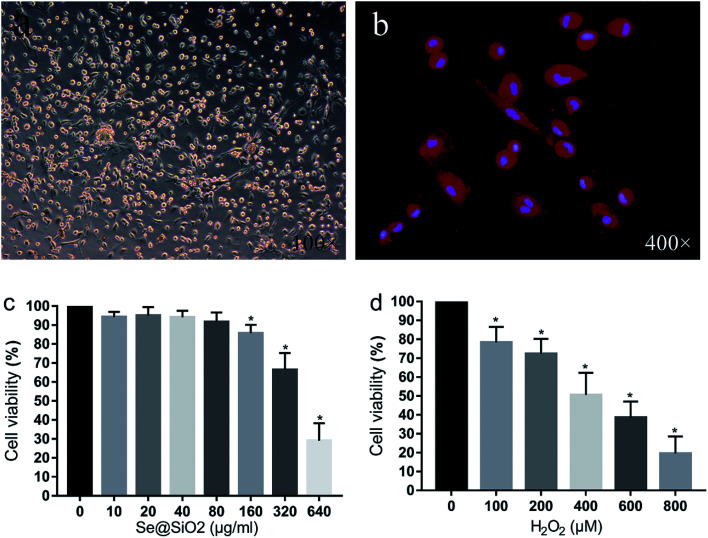
Morphology of the primary microglia and Se@SiO_2_ effects of different concentrations of H_2_O_2_ on microglia viability. (a) Morphology of the primary microglia observed by optical microscopy (100×). (b) Immunohistochemistry of Iba-1 of the primary microglia (400×). (c) The effects of different concentrations of Se@SiO_2_ on primary microglia viability. (d) The effects of different concentrations of H_2_O_2_ on microglia viability. Data are expressed as mean ± SD, *P < 0.05 *vs.* 0 group (*n* = 6).

After 24 h mixed culture, adherent cells showed light transmitting microglia, astrocytes, neurons and some red blood cells. Cultured glial cells began stratifying after 7–9 days, with astrocytes and neurons in the lower layer, and semi-adherent, refractive and round microglia and a small amount of oligodendrocytes in the upper layer. The microglia separated by constant temperature shaking method were round, refractive and uniformly sized. After 24 h inoculation of the purified microglia, cells adhered to the wall and became irregular, spindle shaped, branched or spider-like, without obvious proliferation. Immunofluorescence staining with microglia specific antibody Iba-1 showed that the positive rate of the primary microglia was over 95%, indicating that the purity of the primary microglia was high (Fig. 2).

### Se@SiO_2_ biocompatibility test and the median lethal H_2_O_2_ concentration in primary microglia *in vitro*

As shown in Fig. 2c, the viability of the primary microglia was insignificantly affected when the concentration of Se@SiO_2_ was lower than 80 μg ml^−1^. When the solubility was higher than 80 μg ml^−1^, obvious toxicity could be observed, indicating that the porous Se@SiO_2_ nanocomposites had good biocompatibility. Cell viability was detected by CCK-8 after application of different concentrations of H_2_O_2_ (0, 100, 200, 400, 600, 800 and 1000 μM) to the primary microglia for 24 h (Fig. 2). The viability of the 7 groups were significantly different, and cytotoxicity was positively correlated with the H_2_O_2_ concentration. When the concentration of H_2_O_2_ was 400 μM, the viability of the microglia decreased by about 50%, which was the half lethal dose of microglia and used as the toxicity condition for microglia in the following test (Fig. 2).

### Se@SiO_2_ reduces H_2_O_2_-induced microglia cytotoxicity, oxidative stress injury and release of inflammatory factors *in vitro*

**Fig. 3:**
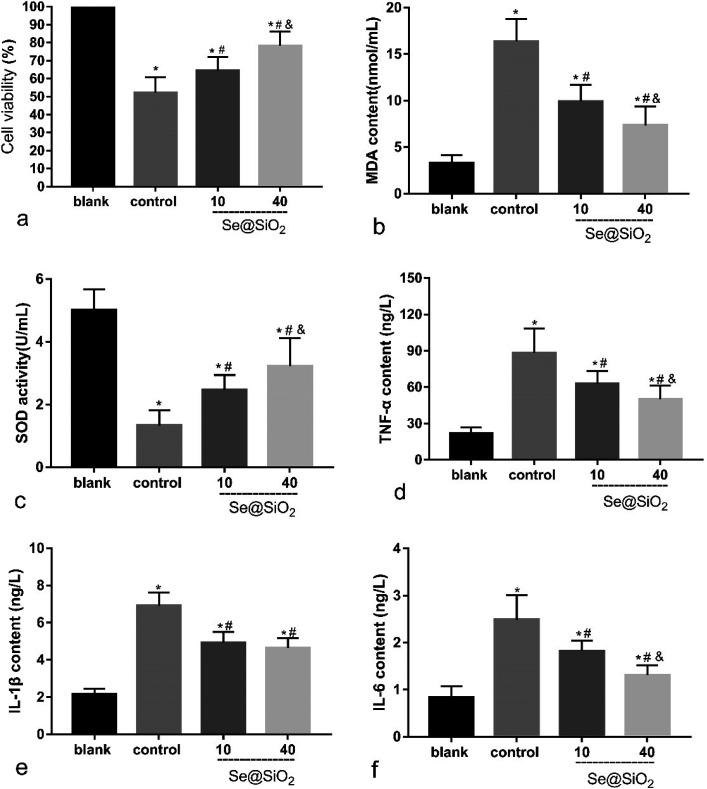
Se@SiO_2_ reduces H_2_O_2_-induced microglia cytotoxicity, oxidative stress injury and release of inflammatory factors. Date are expressed as mean ± SD, *P < 0.05 *vs.* sham group. ^#^*P* < 0.05 *vs.* control group, ^&^*P* < 0.05 *vs.* 10 μg ml^−1^ group (*n* = 6).

Based on the median lethal H_2_O_2_ concentration of the primary microglia, 400 μM was used as a toxicity concentration. The results of CCK-8 showed that Se@SiO_2_ could reduce the cytotoxicity of H_2_O_2_ to primary microglia markedly, and the survival rate of 40 μg ml^−1^ group was significantly higher than that of 10 μg ml^−1^ group (Fig. 3a, *P* < 0.05), indicating that the higher the concentration of Se@SiO_2_, the stronger the ability to reduce cytotoxicity. MDA and SOD assays showed that Se@SiO_2_ could significantly reduce oxidative stress in microglia after H_2_O_2_ toxicity, and the level of oxidative stress in 40 μg ml^−1^ group was significantly lower than that in 10 μg ml^−1^ group (Fig. 3b and c, *P* < 0.05). The results of ELISA showed that the concentration of TNF-α, IL-1β and IL-6 in the supernatant of microglia demonstrated that Se@SiO_2_ could significantly reduce the intensity of inflammatory reaction of microglia after H_2_O_2_ toxicity, and the level of inflammatory reaction in 40 μg ml^−1^ group was significantly lower than that in 10 μg ml^−1^ group (Fig. 3d–f, *P* < 0.05).

### Se@SiO_2_ enhanced axonal regeneration and improved rat hind-limb motor function after SCI

**Fig. 4:**
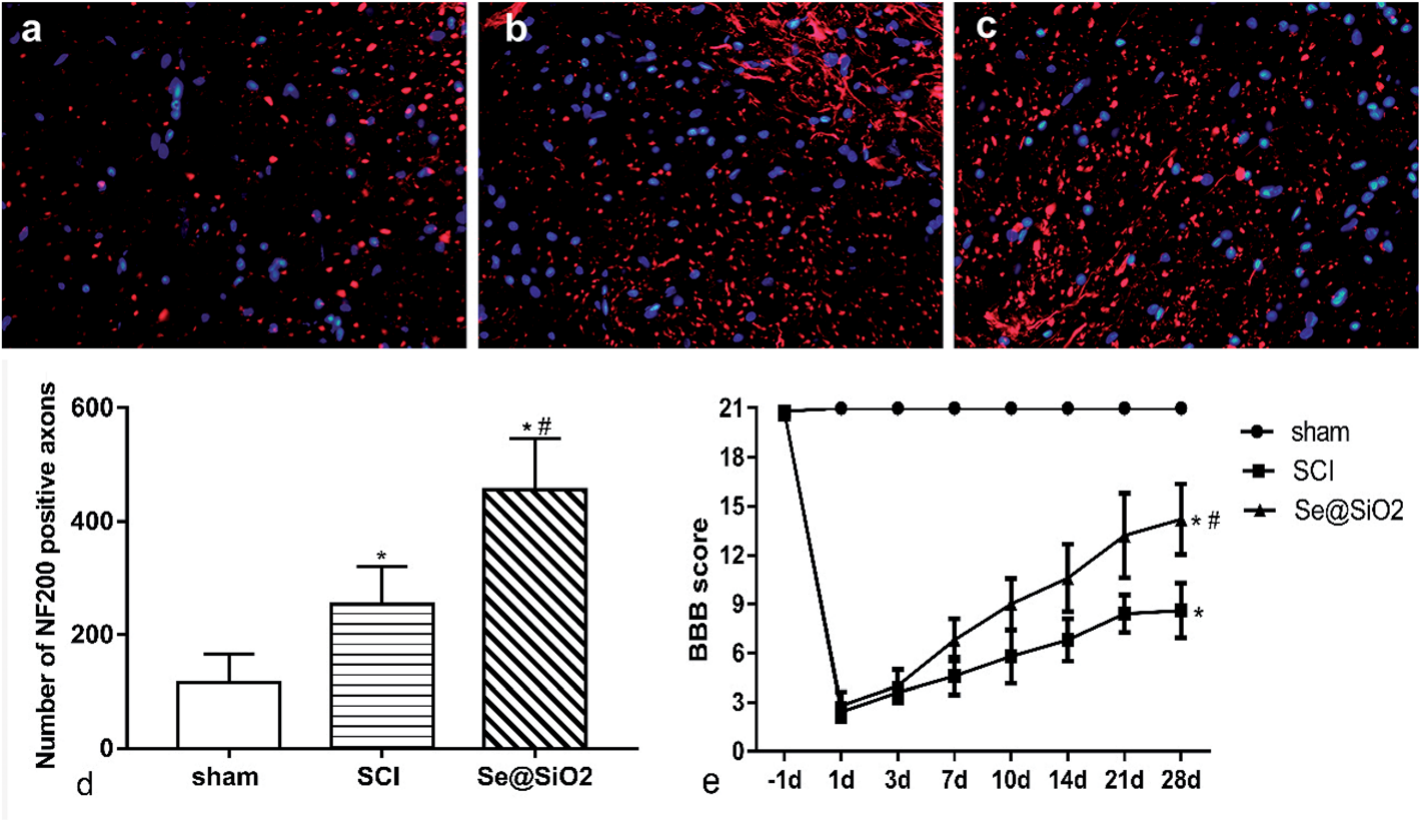
Se@SiO_2_ enhanced axonal regeneration and BBB scoring. (a)–(c) represent NF200 positive axons in the sham, SCI and Se@SiO_2_ groups 28 d after SCI (400×). (d) shows the quantitative data for NF200 positive axons. (e) shows the BBB scoring after SCI. Data are expressed as mean ± SD. *P < 0.05 *vs.* sham group, ^#^*P* < 0.05 *vs.* SCI group (*n* = 5).

BBB scores and axonal regeneration were used to evaluate the effect of Se@SiO_2_ on functional recovery. It was found that the BBB scores increased progressively in both SCI and Se@SiO_2_ groups over time, indicating natural nerve recovery after SCI in rats. Rats receiving Se@SiO_2_ had significantly higher BBB scores than those in SCI group (Fig. 4e, *P* < 0.05, *n* = 15). NF200 was used to identify the axonal regeneration in the spinal cord lesion 14 days after SCI. NF200 is the marker of the medium and large neuron, which can indirectly reflect the degree of axonal injury and repair. The NF200 positive axons were distributed in the vicinity of the injury center, and the number was much higher after SCI. The number of NF200 positive axons in the Se@SiO_2_ group was significantly higher than that of the SCI group and sham group (Fig. 4a–d, *P* < 0.05, *n* = 5).

### Histopathological analysis

**Fig. 5:**
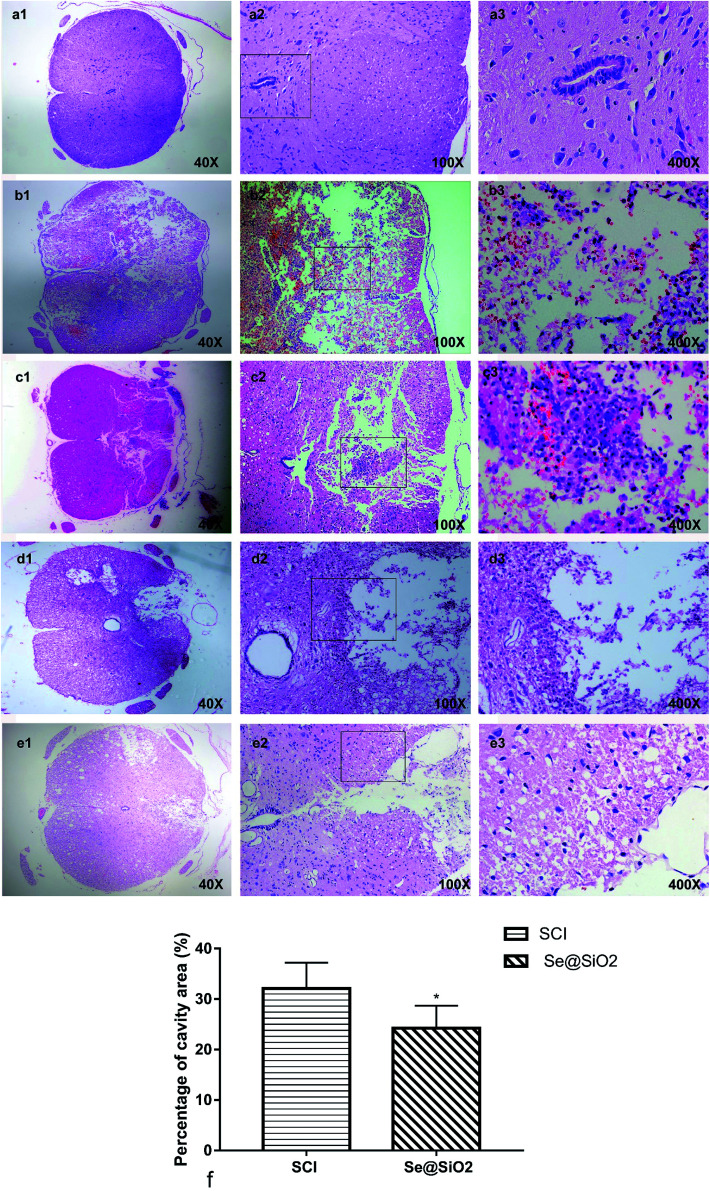
(a) The normal spinal cord. (b) The spinal cord 3 days after SCI in SCI group. (c) The spinal cord in Se@SiO_2_ group 3 days after SCI. (d) The spinal cord 28 days after SCI in SCI group. (e) The spinal cord 28 days after SCI in Se@SiO_2_ group. (f) The quantification percentage of cavity area 28 d after SCI (percentage of cavity area = cavity area/total area × 100%). Data are expressed as mean ± SD (*n* = 5).

A clear boundary between the gray matter and the white matter of the spinal cord tissue was observed in sham group. The outline of nerve cells was clear, the cytoplasm was homogeneous and deeply stained, and the whole tissue structure was intact and clear. The boundary became obscure 3 days after SCI. A large number of necrotic nerve cells, atrophic cytoplasm, degeneration and vacuolar degeneration of cytoplasm, and disorganized arrangement of nerve fibers were observed. There was hemorrhage, liquefaction and inflammatory cell infiltration in the middle of the spinal cord lesion. Cell death, hemorrhage and inflammatory cell infiltration at the spinal cord lesion were attenuated markedly after administration Se@SiO_2_. The neuronal structure was complete; some neuronal cell vacuolar degeneration, a small amount of inflammatory cell infiltration, and nerve fibers arranged in an orderly manner. Fourteen days after SCI, formation of voids was observed in the spinal cord of SCI group. The boundary between the gray matter and the white matter of the spinal cord was unclear, and inflammatory cell infiltration was observed. In contrast, the spinal cavity became smaller and inflammatory cell infiltration was reduced in Se@SiO_2_ group. The quantification percentage of cavity area in the Se@SiO_2_ group was much lower than that in the SCI group 14 days after SCI (Fig. 5, *P* < 0.05, *n* = 5).

### The anti-oxidative and anti-inflammatory effect of Se@SiO_2_*in vivo*

**Fig. 6:**
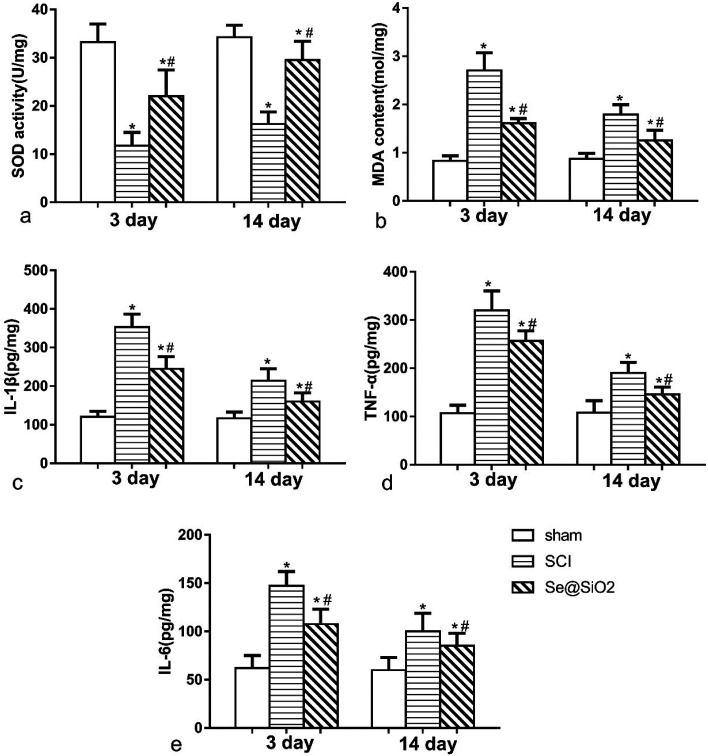
The anti-oxidative and anti-inflammatory effect of Se@SiO_2_*in vivo*. The content of SOD (a), MDA (b), TNF-α (c), IL-1β (d) and IL-6 (e) in sham, SCI and Se@SiO_2_ groups. Data are expressed as mean ± SD. *P < 0.05 *vs.* sham group. ^#^*P* < 0.05 *vs.* SCI group (*n* = 5).

The content of MDA and SOD was detected 3 and 14 days after SCI to determine the level of spinal cord oxidative stress (Fig. 6a and b). Rats with SCI presented with significantly reduced SOD activity and increased MDA content at the lesion site after SCI, especially 3 days after SCI. Compared with SCI group, the level of MDA decreased significantly, and SOD activity increased significantly in Se@SiO_2_ group after 3 and 14 days (*P* < 0.05). Se@SiO_2_ intrathecal injection could reduce the level of oxidative stress of the damaged spinal cord markedly 3 and 14 days after SCI. The contents of TNF-α, IL-1β and IL-6 were detected 3 and 14 days after SCI by ELISA to determine SCI inflammation (Fig. 6c–e). TNF-α, IL-1β and IL-6 were major indicators of inflammation in the spinal cord. Compared with sham group, TNF-α, IL-1β and IL-6 levels in SCI group increased significantly after SCI (*P* < 0.05). However, the TNF-α, IL-1β and IL-6 contents reduced significantly after Se@SiO_2_ treatment (*P* < 0.05). In addition, TNF-α, IL-1β and IL-6 levels decreased markedly in SCI group after 14 days treatment compared with those after 3 days treatment (*P* < 0.05).

### Se@SiO_2_ suppresses microglia activation *in vivo*

**Fig. 7:**
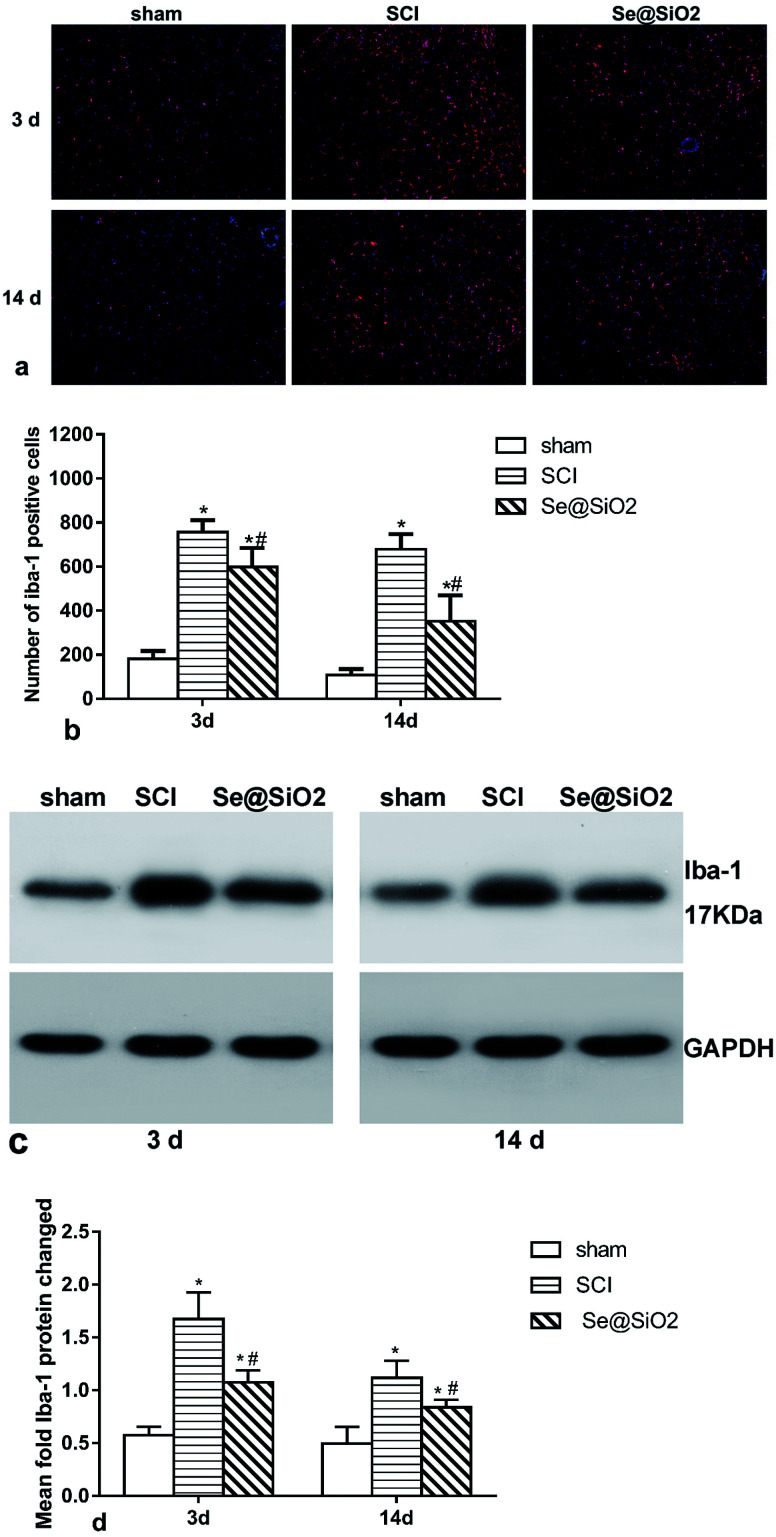
Microglia activation in the spine cord lesion 3 and 14 days after SCI as assayed by western blot and immunofluorescence. (a) and (b) show the marker of the activated microglia Iba-1 as tested by immunofluorescence (100×). (c) and (d) show Iba-1 protein in the spinal lesion site as evaluated by western blot. Data are expressed as mean ± SD, *P < 0.05 *vs.* sham group. ^#^*P* < 0.05 *vs.* SCI group (*n* = 5).

The activation of microglia played a critical role in the pathogenesis of SCI. To study the effect of Se@SiO_2_ on ROS and inflammation, microglia in the damaged spinal cord lesion were tested by immunofluorescence (Fig. 7a and b) and Western blot of Iba-1 protein (Fig. 7c and d). The number of microglia (Iba-1+) and Iba-1 protein increased significantly 3 and 14 days after SCI as compared with sham group (*P* < 0.05), while the number of microglia and Iba-1 protein in Se@SiO_2_ group decreased significantly compared with SCI group 3 and 14 days after SCI. The result of morphological analysis suggested that positive Iba-1 cells were activated microglia after SCI. The activation of microglia after SCI could be inhibited by Se@SiO_2_.

### The anti-apoptotic effect of Se@SiO_2_*in vivo*

**Fig. 8:**
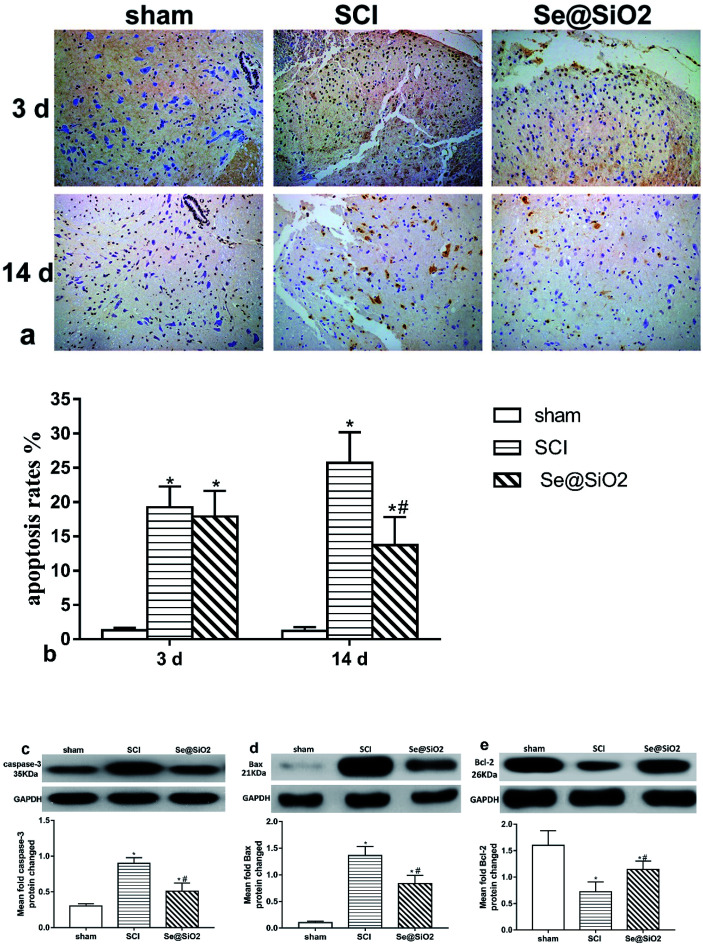
Anti-apoptotic effect of Se@SiO_2_ in SCI investigated by using WB and TUNEL staining. (a) and (b) shows the apoptosis rates tested by TUNEL stained 3 and 14 days after SCI (200×). (c)–(e) shows the caspase-3, Bax and Bcl-2 protein in spinal lesion site evaluated by western blot 14 days after SCI. Data are expressed as mean ± SD, *P < 0.05 *vs.* sham group. ^#^*P* < 0.05 *vs.* SCI group (*n* = 5).

TUNEL and western-blot showed that Se@SiO_2_ exerted an anti-apoptotic effect after SCI. TUNEL staining was used to evaluate the cell apoptosis rate (Fig. 8a and b). The content of caspase-3, Bcl-2 and Bax in the spinal site as detected by western blot (Fig. 8c–e). TUNEL staining showed that the number of apoptosis cells increased significantly in SCI group at 3 and 14 days after SCI, but decreased markedly in Se@SiO_2_ group compared with SCI group at 14 days (*P* < 0.05). Western blot assay shown that SCI increased the caspase-3 and Bax in the spinal lesion site as compared to that in sham group, but markedly decreased the levels of Bcl-2 (*P* < 0.05). Western blot assay also validated that the protein content of caspase-3 and Bax increased significantly in SCI group compared with sham group, while the Bcl-2 decreased significantly 14 days after SCI (*P* < 0.05).

## Discussion

Oxidative stress damage caused by the imbalance of ROS production and the decrease in antioxidant capacity of the cell is thought to initiate and be the main mechanism of multiple neurological diseases such as Alzheimer's disease,^49^ Parkinson's disease,^50^ amyotrophic lateral sclerosis^51^ and traumatic brain and SCI.^52^ Se is one of the essential elements of the human body. At present, 25 kinds of selenoproteins have been found in human proteome, including the glutathione peroxidase family (GPxs), thiored oxidoreductase family (TrxRs), and deiodinase family. In addition to selenoprotein P, other selenoproteins contain a common active center of selenocysteine (Sec). Most Se proteins including GPxs and TrxRs are involved in the regulation of antioxidant defense and redox signaling. Se is also involved in the regulation of other special biological processes, including the biosynthesis of DNA, degradation of faulty folding proteins in the endoplasmic reticulum, regulation of redox transcription factors, apoptosis and immunoregulation, and transportation and storage of Se.^53^

In this experiment, we investigated the anti-oxidative effect of porous Se@SiO_2_ nanocomposites on microglia during SCI *in vivo* and *in vitro*. The newly prepared porous Se@SiO_2_ nanocomposites are nano-modified in order to reduce the risk of nano-Se toxicity and improve the biological activity. Using porous, silica, nanocarriers to control the release of nano-Se can provide an effective concentration of Se for a long time and improve the treatment efficacy. Our *in vitro* experiment showed that porous Se@SiO_2_ nanocomposites had favorable biocompatibility (Fig. 2). Knowing that exogenous H_2_O_2_ can react with intracellular ions (iron or copper) to produce highly toxic ROS causing DNA damage and cell death, we used H_2_O_2_ in this study to produce an oxidative stress injury model.^16^ We found that porous Se@SiO_2_ nanocomposites could reduce the ROS damage effectively against H_2_O_2_ induced microglia activation and release of inflammatory factors (Fig. 3). Microglia were characterized by high oxygen consumption, strong metabolism, easiness to produce oxygen free radicals, and susceptibility to ROS.^54^*In vivo*, we measured the content of microglia-specific protein Iba-1 by western blot and immunofluorescence at different time points at the injury site of SCI in rats (Fig. 7) and found that Se could effectively reduce microglial hyperactivity in SCI-modeled rats. In addition, Se could markedly reduce the degree of oxidative stress and inflammatory reaction (Fig. 6) of the injured segments *in vivo*. Porous Se@SiO_2_ nanocomposites could significantly suppress microglia activation related to oxidative stress and inflammation after SCI, improve the hind-limb neurological function (Fig. 4) and reduce apoptosis *via* the caspase-3 pathway (Fig. 8) in the rats after SCI.

Se played a role in inhibiting microglial activation by significantly reducing the production of ROS and the release of inflammatory factors caused by H_2_O_2_. The specific molecular mechanism of Se in cells is the focus of current research. Studies have shown that Se can reduce the cellular oxidative stress level by increasing the expression or function of the intracellular GPxs and the TrxRs.^18^ Studies have also shown that Se can inhibit the activation of 3 kinase/protein kinase B (PI3K/AKT) and extracellular regulatory kinase (ERK) signaling pathways, and reduce the calcification of vascular smooth muscle cells induced by oxidative stress. Se can weaken the oxidative stress of activated endoplasmic reticulum and reduce apoptosis of smooth muscle cells.^55^ Se can reduce the infarct size and the content of TNF-α after ischemia by inhibiting the NF-κB pathway.^56^ However, the specific molecular mechanisms involved in the inhibition of Se on microglial activation remain unclear, and need further studies.

Recent advances in nanoengineering have provided promising alternatives for the design of biocompatible micro–nano and nanocarriers for the controlled release of therapeutic agents to target CNS cells.^57–60^ Some of the most promising nanostructures for delivery of therapeutic agents to promote SCI recovery include polymer nanoparticles,^61^ surface-functionalized iron oxide nanomagnets,^62^ carbon/metal nanotubes,^63^ nanocomposites,^64^ and micro or nanofabricated nerve devices.^65,66^ The ability to precisely control dosing regimens and the implementation of specific targeting strategies may promote the recovery after SCI and reduce toxic side effects. In order to solve the problem of the Se safety range,^27,28^ this experiment used the porous Se@SiO_2_ nano composites,^36^ a new type of material that controls the release of Se. Some recent studies demonstrated that porous Se@SiO_2_ nanocomposites could inhibit steroid-induced osteonecrosis of the femoral head by inhibiting oxidative stress.^42^ The advantage of this material is that the nano-modified Se elements could not only significantly reduce the toxicity risk of nano Se^37,38^ but increase the biological activity of se.^39,40^ The use of porous and silica nanocarriers to control the release of nano-Se could achieve a long-term effective concentration of Se and improve the treatment efficacy.^42^ Compared with normal Se nanoparticles, Se in the Se@SiO_2_ nanocomposites was limited by SiO2. Accompanying the entrance of PVP into an aqueous solution, trace Se can be released into the solution.^36^ Our *in vitro* experiment showed that the porous silica nanocarrier was relatively stable, had good biocompatibility (Fig. 2), and the loaded Se elements could be continuously released to pH 7.4 0.01 M PBS (Fig. 1). In addition, the remarkable biocompatibility of porous Se@SiO_2_ nanocomposites could reduce ROS damage effectively *in vitro*.

Se could inhibit apoptosis of the damaged segment induced by SCI by inhibiting the caspase-3 pathway *in vivo* (Fig. 8). Experiments had shown that *in situ* injection of Se can inhibit ROS-mediated apoptotic neural precursor cell death in traumatic brain injury.^26^ Our study showed that the inhibitory effect of intrathecal injection of porous Se@SiO_2_ nanocomposites on apoptosis was especially pronounced 14 days after SCI. On the one hand, Se may probably inhibit apoptosis in the spinal cord by inhibiting the intensity of oxidative stress and inflammatory response associated with excessive activation of microglia. On the other hand, it may be because of the direct enhancement of neuronal cell resistance. The specific mechanism underlying the anti-apoptosis effect of Se needs further study.

## Conclusion

Our current findings demonstrated that porous Se@SiO_2_ nanocomposites may prove to be a promising strategy for protection against SCI by suppressing microglia-mediated ROS and apoptosis. Porous Se@SiO_2_ nanocomposites are a new, stable and effective material with great potential in clinical treatment of SCI.

## Author contribution statement

Weiheng Wang, Xiaodong Huang and Yongxing Zhang wrote the manuscript and did the main research. Guoying Deng and Chunquan Fan prepared the figures. Se@SiO_2_ used in this study was invented and provided by Xijian Liu. Xiaojian Ye, Jiangming Yu and Yanhai Xi designed and supported this experiment.

## Conflicts of interest

The authors declare that there is no conflict of interests regarding the publication of this paper.

## Supplementary Material
